# Evaluating Foundational Data Quality in the National Patient-Centered Clinical Research Network (PCORnet®)

**DOI:** 10.5334/egems.199

**Published:** 2018-04-13

**Authors:** Laura Goettinger Qualls, Thomas A. Phillips, Bradley G. Hammill, James Topping, Darcy M. Louzao, Jeffrey S. Brown, Lesley H. Curtis, Keith Marsolo

**Affiliations:** 1 Duke Clinical Research Institute, US; 2 Harvard Pilgrim Health Care Institute, US; 3 Cincinnati Children’s Hospital Medical Center, US

**Keywords:** electronic health records, quality improvement, patient-centered care, distributed research networks, data quality

## Abstract

**Introduction:**

Distributed research networks (DRNs) are critical components of the strategic roadmaps for the National Institutes of Health and the Food and Drug Administration as they work to move toward large-scale systems of evidence generation. The National Patient-Centered Clinical Research Network (PCORnet®) is one of the first DRNs to incorporate electronic health record data from multiple domains on a national scale. Before conducting analyses in a DRN, it is important to assess the quality and characteristics of the data.

**Methods:**

PCORnet’s Coordinating Center is responsible for evaluating foundational data quality, or assessing fitness-for-use across a broad research portfolio, through a process called data curation. Data curation involves a set of analytic and querying activities to assess data quality coupled with maintenance of detailed documentation and ongoing communication with network partners. The first cycle of PCORnet data curation focused on six domains in the PCORnet common data model: demographics, diagnoses, encounters, enrollment, procedures, and vitals.

**Results:**

The data curation process led to improvements in foundational data quality. Notable improvements included the elimination of data model conformance errors; a decrease in implausible height, weight, and blood pressure values; an increase in the volume of diagnoses and procedures; and more complete data for key analytic variables. Based on the findings of the first cycle, we made modifications to the curation process to increase efficiencies and further reduce variation among data partners.

**Discussion:**

The iterative nature of the data curation process allows PCORnet to gradually increase the foundational level of data quality and reduce variability across the network. These activities help increase the transparency and reproducibility of analyses within PCORnet and can serve as a model for other DRNs.

## Introduction

Distributed research networks (DRNs) are critical components of the strategic roadmaps for the National Institutes of Health and the Food and Drug Administration as they work to move toward large-scale systems of evidence generation [1,2]. The promise and sustainability of these systems hinges on the ability to extract usable, high-quality data from electronic health records (EHRs), health insurance claims, and other sources that are fit to support translational, interventional, and observational research initiatives. Currently, there are several active DRNs in the United States, including the Sentinel Initiative [3,4], the Health Care Systems Research Network (HCSRN) [5,6], the National Institutes of Health’s Health Care Systems Research Collaboratory Distributed Research Network [7], and the National Patient-Centered Clinical Research Network (PCORnet®; www.pcornet.org) [8,9]. In most DRNs, data are housed locally and standardized to a common data model (CDM). Analysis programs, or queries, are distributed to the network partners, and results are sent back to the requestor [10,11].

PCORnet is an innovative initiative funded by the Patient-Centered Outcomes Research Institute (PCORI). The goal of PCORnet is to improve the nation’s capacity to conduct health research, particularly comparative effectiveness research, by creating a large, highly representative network for conducting clinical outcomes research. PCORnet is structured as a “network of networks” that includes 13 Clinical Data Research Networks (CDRNs), 20 Patient-Powered Research Networks (PPRNs), and 2 Health Plan Research Networks (HPRNs) [12]. PCORnet’s Coordinating Center is managed by Duke Clinical Research Institute, Genetic Alliance, and Harvard Pilgrim Healthcare Institute. CDRN partner institutions include hospitals, ambulatory care clinics, health plans, integrated health systems, and public health providers. These network partners contribute data from EHRs, billing systems, registries, health insurance claims, and other sources.

PCORnet is one of the first DRNs to incorporate EHR data from multiple domains on a national scale. Data are stored in DataMarts which conform to the PCORnet Common Data Model [13]. CDRNs maintain two instances of each DataMart: one using a relational database management system (RDBMS), and another as SAS® datasets or views. The SAS version of the CDM is used to answer PCORnet research queries and is subject to the data curation process described in this paper. The RDBMS version of the DataMart is intended to be refreshed more frequently and is reserved for basic questions of study feasibility. CDRNs have adopted a variety of network models, ranging from centralized (data from all institutions are stored in one DataMart) to distributed (each institution has one DataMart) to a hybrid approach (data from several institutions is centralized into one DataMart while other institutions have their own DataMart).

Before conducting analyses in a DRN, it is important for researchers to first understand the quality and characteristics of the available data [14,15]. DRNs have developed a variety of processes to evaluate the quality of the data in their networks [14,16,17,18]. In PCORnet, data quality assessments encompass a broad range of activities including those conducted by individual institutions, the CDRNs [19], and the Coordinating Center. Many network partners also participate in other DRNs and benefit from the data quality assessment activities of those DRNs. Network-wide data quality assessment is particularly important since PCORnet represents one of the first times that EHR data have been evaluated for research use at a national scale. PCORnet’s Coordinating Center is responsible for evaluating foundational data quality, or assessing fitness-for-use across a broad research portfolio, through a process called data curation. The data curation process was designed to be pragmatic, scalable, data-driven, and transparent [20]. In this paper, we describe the development, implementation, and results of the first data curation cycle and improvements made in subsequent cycles.

## Methods

### Overview

**Figure 1:**
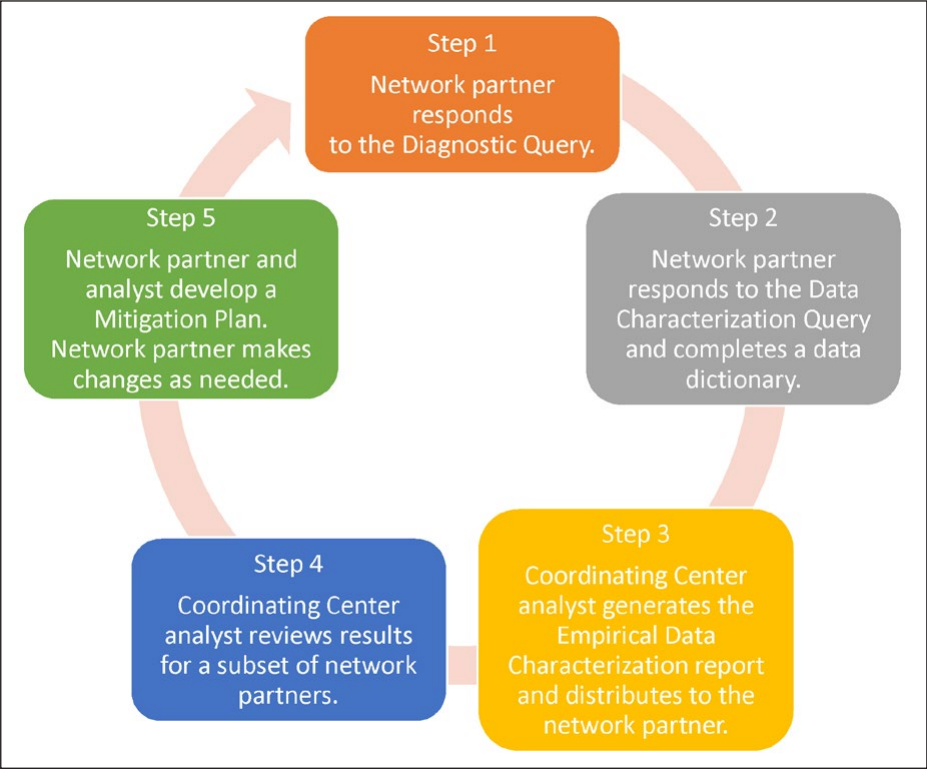
Data Curation Cycle.

The first cycle of data curation focused on six domains in the PCORnet CDM: demographics, diagnoses, encounters, enrollment, procedures, and vitals. We designed a five-step data curation process (Figure 1). All network partners were expected to participate in Steps 1 through 3, and a subset were expected to participate in Steps 4 and 5. The cycle was planned to run from January through June 2016 and include up to 50 of the 82 CDRN DataMarts in Steps 4 and 5.

The process for a network partner who completed all steps of the data curation cycle was as follows. First, the network partner responded to a diagnostic query, which evaluated table-level conformance to the CDM. Second, the network partner completed a data dictionary and executed a data characterization query. The data dictionary captured information about each DataMart’s technical infrastructure; data provenance; implementation status for each table and field (implemented, deferred, not feasible, or not available); and notes about the extract, transform, and load process. The data characterization query generated table-level and field-level frequencies, descriptive statistics, and cross-tabulations. Third, using the data characterization query output, analysts at the Coordinating Center created a summary report called the Empirical Data Characterization Report and distributed it to the network partner. Fourth, analysts at the Coordinating Center reviewed the data characterization query output, the data dictionary, and the Empirical Data Characterization Report, and identified topics for discussion with the network partner. Fifth, the analyst discussed his or her observations with the network partner. If opportunities for improvement were identified, they were documented in a mitigation plan. Improvements could be made within the current cycle or deferred to a future cycle. Network partners who made changes to their data (“refreshed” the DataMart) reran the data characterization query and received a new Empirical Data Characterization Report.

### Data Quality Checks

We created data quality measures for data domains that have been well-characterized in other networks or are widely used in health services or comparative effectiveness research. Measures were developed based on early experiences in analyzing data from 11 DataMarts using processes informed by the Food and Drug Administration’s Sentinel Data Quality Review and Characterization Programs [21], and were refined based on feedback received from network partners and other stakeholders. We implemented 13 data quality checks, or rules, pertaining to three categories of data quality: data model conformance (7 checks), data plausibility (2 checks), and data completeness (4 checks) [22]. Each of these data quality checks was applied to more than one data element or table, resulting in 314 discrete data quality measures (Table 1). Data model conformance checks 1.01 through 1.04 were applied to all tables and fields, whereas all remaining checks were applied only to the six domains of interest (demographics, diagnoses, encounters, enrollment, procedures, and vitals).

**Table 1:** Data Quality Checks.

Category	Check	Description	Number of measures

Data Model Conformance	1.01	Required tables are not present	15 tables
	1.02	Expected tables are not populated	7 tables
	1.03	Required fields are not present	188 fields
	1.04	Fields do not conform to CDM specifications for data type, length, or name	188 fields
	1.05	Tables have primary key definition errors	7 tables
	1.06	Fields contain values outside of CDM specifications	29 fields
	1.07	Fields have non-permissible missing values	25 fields
Data Plausibility	2.01	More than 5% of records have future dates	7 fields
	2.02	More than 20% of records fall into the lowest or highest categories of age, height, weight, diastolic blood pressure, systolic blood pressure.	5 fields
Data Completeness	3.01	The average number of diagnoses records per encounter is less than 1.0 for ambulatory, inpatient, emergency department, or ED-to-inpatient encounters.	4 measures
	3.02	The average number of procedure records per encounter is less than 1.0 for ambulatory, inpatient, emergency department, or ED-to-inpatient encounters.	4 measures
	3.03	More than 5% of records have missing or unknown values for the following fields: birth date; sex; diagnosis code type, procedure code type, and vital source.	5 fields
	3.04	More than 15% of records have missing or unknown values for the following fields: race, discharge disposition (institutional encounters only), and principal diagnosis code (institutional encounters only).	3 fields

### Analysis Programs

We developed three analysis programs, or queries, to measure the quantifiable characteristics of each DataMart using SAS® 9.3 software: the Diagnostic Query, the Data Characterization Query, and the Empirical Data Characterization Query. Programs were designed to be easily executed by analysts who had little knowledge of SAS software and across a wide range of technical configurations and DataMart sizes. The Diagnostic Query produced one table and an exception report. The Data Characterization Query generated 77 output tables. Both queries produced SAS datasets and a PDF document which could be easily reviewed by investigators and other stakeholders. The Empirical Data Characterization Query compiled the data characterization query results into a normalized dataset and produced the Empirical Data Characterization report. The Empirical Data Characterization Report summarized key descriptive information, graphically displayed longitudinal trends, and displayed results for the data quality measures, with exceptions being highlighted (Supplemental Material 1).

## Results

Network partners were expected to respond to queries within 14 days. The Diagnostic Query was distributed to network partners in January 2016. Approximately 62 percent of partners (n = 51/82) responded within the expected turnaround time. Turnaround times ranged from 0 to 58 days, with a median of 11 days. Delays were primarily related to technical challenges in correctly implementing the SAS instantiation of the DataMart. The Data Characterization Query was distributed upon completion of the Diagnostic Query, and 70.7 percent (n = 58/82) responded on time. Turnaround times ranged from 0 to 57 days, with a median of 11 days. Delays in responding to the Data Characterization Query were primarily related to user errors in executing the query, insufficient memory or storage, and needing time to correct errors. Analysts at the Coordinating Center created Empirical Data Characterization Reports for network partners beginning in March 2016.

To complete cycle 1, network partners were required to correct any data model conformance errors uncovered through data curation and to meet two additional requirements established by PCORI: to sign a data sharing agreement with the Coordinating Center and to capture diagnosis and procedure data for at least half of the patients who had health care encounters. Data curation cycle 1 was scheduled to end in June 2016, but was extended through September 2016. Network partners at 64 of the 82 DataMarts (78.0 percent) completed cycle 1.

Upon running the data characterization program for the first time, 65.6 percent of the network partners that completed cycle 1 (42/64) had one or more data model conformance error. During the cycle, 82.8 percent (53/64) refreshed the DataMart one or more times to improve data quality. The number of refreshes per DataMart ranged from one to four. Network partners at 53 DataMarts participated in Steps 4 and 5 of the data curation cycle. Analysts at the Coordinating Center reviewed the data dictionaries, data characterization query output, and Empirical Data Characterization Reports to look for unusual patterns in the trend charts, exceptions to data quality measures, discrepancies between the data dictionary documentation and the data, and contextually implausible results (e.g., an average height of 5 feet for a pediatric population). Analysts identified 573 items for discussion with network partners. Discussion items were classified by topic as follows: 26.0 percent on extract, transform, and load practices (n = 149); 24.1 percent on data plausibility (n = 138); 20.1 percent on data completeness (n = 115); 15.0 percent on data conformance (n = 86); 12.0 percent on provenance (n = 69); and 2.8 percent on other topics (n = 16).

Analysts and network partners communicated via telephone and e-mail to discuss these findings and develop a mitigation plan if necessary. Changes made by network partners as a result of the data curation conversations included the following: remediating data model errors (e.g., values outside of specifications, incorrect source data mapping, referential integrity problems); reclassifying data (e.g., distinguishing telephone consults from face-to-face encounters); adding data sources (e.g., billing data), data domains (e.g., vital signs), or sub-domains (e.g., infused medications in the procedures table); correcting diagnosis and procedure code type misclassifications (e.g., Tenth International Classification of Diseases-Clinical Modification codes misclassified as Ninth International Classification of Diseases-Clinical Modification codes); removing data that were not part of the model scope (e.g., administrative encounters, appointments); and eliminating duplicate records. The number of DataMarts with exceptions to the data quality checks decreased between the baseline and final refresh (Table 2). Specifically, the number of DataMarts with exceptions decreased for all seven data model conformance checks, for one data plausibility check (three of 12 measures), and for all four data completeness checks (14 of 16 measures). Descriptive statistics for data completeness measures are shown in Table 3. Notable improvements include an increase in the median diagnoses per inpatient encounter from 6.92 to 9.62, an increase in the median procedures per inpatient encounter from 4.42 to 10.14, and a decrease in the median percentage of institutional encounters with missing or unknown discharge disposition from 24.41 percent to 3.48 percent.

**Table 2:** Data Quality Results, Number and Percentage of DataMarts* with Data Check Exceptions.

Category	Check	Description	Baseline Refresh	Final Refresh

Data Model Conformance	1.01	Required tables are not present	2/64 (3.0%)	0/64 (0.0%)
	1.02	Expected tables are not populated	1/64 (1.6%)	0/64 (0.0%)
	1.03	Required fields are not present	1/64 (1.6%)	0/64 (0.0%)
	1.04	Fields do not conform to CDM specifications for data type, length, or name	2/64 (3.1%)	0/64 (0.0%)
	1.05	Tables have primary key definition errors	11/64 (17.2%)	0/64 (0.0%)
	1.06	Fields contain values outside of CDM specifications	17/64 (26.6%)	0/64 (0.0%)
	1.07	Fields have non-permissible missing values	13/64 (20.3%)	0/64 (0.0%)
Data Plausibility	2.01	More than 5% of records have future dates:		
		Birth Date	0/64 (0.0%)	0/64 (0.0%)
		Admit Date	0/64 (0.0%)	0/64 (0.0%)
		Discharge Date, institutional encounters	0/60 (0.0%)	0/59 (0.0%)
		Procedure Date	0/54 (0.0%)	0/62 (0.0%)
		Enrollment Start Date	0/64 (0.0%)	0/64 (0.0%)
		Enrollment End Date	0/64 (0.0%)	0/64 (0.0%)
		Measure Date	0/62 (0.0%)	0/62 (0.0%)
	2.02	More than 20% of records fall into the lowest or highest categories of age, height, weight, diastolic blood pressure, systolic blood pressure:		
		Age	0/62 (0.0%)	0/63 (0.0%)
		Height	3/60 (5.0%)	0/60 (0.0%)
		Weight	3/62 (4.8%)	0/62 (0.0%)
		Diastolic blood pressure	5/60 (8.3%)	1/60 (1.7%)
		Systolic blood pressure	1/60 (1.7%)	1/60 (1.7%)
Data Completeness	3.01	The average number of diagnoses records per encounter is less than 1.0 for ambulatory, inpatient, emergency department, or emergency department to inpatient encounters.		
		Ambulatory	7/60 (11.7%)	2/64 (3.1%)
		Inpatient	8/53 (15.1%)	1/58 (1.7%)
		Emergency department	7/59 (11.9%)	0/59 (0.0%)
		Emergency department to inpatient	1/14 (7.1%)	0/20 (0.0%)
	3.02	The average number of procedure records per encounter is less than 1.0 for ambulatory (AV), inpatient, emergency department, or emergency department to inpatient encounters.		
		Ambulatory	23/59 (39.0%)	13/64 (20.3%)
		Inpatient	9/52 (17.3%)	11/58 (19.0%)
		Emergency department	18/58 (31.0%)	0/59 (0.0%)
		Emergency department to inpatient	1/14 (7.1%)	1/20 (5.0%)
	3.03	More than 5% of records have missing or unknown values for the following fields: birth date; sex; diagnosis code type, procedure code type, and vital source.		
		Birth date	2/64 (3.1%)	1/64 (1.6%)
		Sex	1/64 (1.6%)	0/64 (0.0%)
		Diagnosis code type	2/64 (3.1%)	0/64 (0.0%)
		Procedure code type	16/63 (25.4%)	14/64 (21.9%)
		Vital source	12/62 (19.4%)	7/62 (11.3%)
	3.04	More than 15% of records have missing or unknown values for the following fields: race, discharge disposition (institutional encounters only), and principal diagnosis code (institutional encounters only).		
		Race	44/64 (68.8%)	44/64 (68.8%)
		Discharge disposition (institutional encounters only)	31/64 (48.4%)	24/59 (40.7%)
		Principal diagnosis code (institutional encounters only)	17/64 (26.6%)	11/59 (18.6%)

*The number of DataMarts varies by measure because of the data available in each DataMart. The number of DataMarts for a given measure may vary between the baseline and final refresh if network partners added, removed, or reclassified the data in the DataMart.

**Table 3:** Data Quality Results, Descriptive Statistics for Data Completeness Checks.

	Baseline Refresh				Final Refresh			

	DataMarts*	Min	Median	Max	DataMarts*	Min	Median	Max

Data Check 3.01 Diagnosis records per encounter, N								
Ambulatory encounters	60	0.00	2.17	100.16	64	0.77	2.08	6.14
Inpatient encounters	59	0.00	6.92	46.99	59	1.11	9.62	45.97
Emergency Department encounters	53	0.00	3.26	12.54	58	0.00	3.37	12.62
ED to inpatient encounters	14	0.55	14.19	51.24	20	2.84	15.47	77.41
**Data Check 3.02 Procedure records per encounter, N**								
Ambulatory encounters	59	0.00	1.07	166.10	64	0.00	1.56	8.32
Inpatient encounters	58	0.00	4.42	173.90	59	0.00	10.14	173.18
Emergency Department encounters	52	0.00	1.36	16.66	58	0.00	3.47	16.66
ED to inpatient encounters	14	0.41	20.77	158.62	20	0.45	41.54	159.05
**Data Checks 3.03 and 3.04 Missing or unknown values, % of records**								
Birth date	64	0.00	0.00	82.51	64	0.00	0.00	8.51
Sex	64	0.00	0.04	6.00	64	0.00	0.04	5.82
Diagnosis type	64	0.00	0.00	17.85	64	0.00	0.00	1.82
Procedure type	63	0.00	0.00	100.00	64	0.00	0.00	100.00
Vital source	62	0.00	0.00	100.00	62	0.00	0.00	100.00
Race	64	0.67	27.20	86.43	64	0.69	21.94	86.09
Discharge disposition, institutional encounters	59	0.00	24.41	100.00	59	0.00	3.48	100.00
Principal diagnoses, institutional encounters	58	0.00	0.00	100.00	59	0.00	0.00	100.00

*The number of DataMarts varies by measure because of the data available in each DataMart. The number of DataMarts for a given measure may vary between the baseline and final refresh if network partners added, removed, or reclassified the data in the DataMart. ED = emergency department.

During the discussions with the network partners, analysts also identified topics that could benefit from additional Coordinating Center guidance and/or changes to the CDM. These topics included uncertainty on whether to include certain records in the procedures table (e.g., laboratory test orders billed as procedures), variability in how to classify professional consults in an institutional care setting, poor ability to differentiate the subtypes of Current Procedural Terminology and Healthcare Common Procedure Coding System procedure codes, and variability in how source data were mapped to standard terminologies. Finally, analysts identified data quality measures in which further improvement was not expected (e.g., race data were highly missing prior to the advent of Meaningful Use standards) [23].

## Discussion

The data curation process helped network partners make significant improvements in foundational data quality. Notable improvements included the elimination of data model conformance errors; a decrease in implausible height, weight, and blood pressure values; and more complete data for key analytic variables such as diagnoses, procedures, and discharge disposition.

Based on the experiences during PCORnet’s first data curation cycle, the Coordinating Center implemented the following changes to increase efficiency, transparency, and collaboration:

- We modified the analysis programs to run as a self-contained package, so the diagnostic query, data characterization query, and Empirical Data Characterization report are now all produced by the network partners, facilitating partner review.
- We classified the data quality checks as required or investigative. Exceptions to required data checks, such as data model conformance errors, must be fixed, while exceptions to investigative data checks are permissible but must be explained and classified as remediable or a limitation of the source data.
- We summarized data quality check exceptions at the beginning of the Empirical Data Characterization report to facilitate the review and analysis of these data.
- To streamline data entry and analysis, we collected and managed data dictionaries using REDCap electronic data capture tools hosted at Duke Clinical Research Institute [24].
- We incorporated learnings from the data curation process into v3.1 of the CDM [13].
- We developed Implementation Guidance to mitigate the variability in how network partners map their source data into the CDM. The Implementation Guidance addresses issues at three levels: 1) general – issues that apply to more than one data domain, such as dealing with updated or corrected source values across refreshes; 2) table-level – guidance that is specific to a given data domain, such as inclusion of additional laboratory results; and 3) field-level – guidance that is specific to a single field within the CDM, such as preferred ordering strategy for choosing an RxNorm concept-unique identifier. The Implementation Guidance will continue to be updated over time to incorporate best practices or mapping strategies for issues raised by partners or uncovered through analysis.
- We revised how the Coordinating Center communicates with network partners. One-on-one conversations were resource-intensive and ineffective at disseminating information on common issues and mitigation strategies. Therefore, we implemented network-wide discussion forums to review data curation results and discuss common topics or themes, such as strategies for assigning LOINC (Logical Observation Identifiers Names and Codes; https://loinc.org) codes to laboratory results or assigning RxNorm Concept Unique Identifiers [25] to a medication order. All network partners are invited to attend, and those that have developed best practices are recruited to help lead discussions. Discussion forums are recorded and posted on a web-based collaboration space for subsequent viewing. Findings from these forums inform future refinement of the CDM, Implementation Guidance, and data quality measures.

At the time of this writing, PCORnet is in its fourth cycle of data curation. By implementing the changes described above, the Coordinating Center was able to curate data from all DataMarts beginning in November 2016, and to increase the frequency of data curation from semi-annually to quarterly beginning in January 2017. Data curation activities now encompass 7 additional domains: lab results, medications, death, cause of death, patient-reported outcomes, and problem lists. Data quality checks have been refined and expanded to include additional measures of data model conformance, data plausibility, and data completeness. Examples of new checks include the percentage of quantitative lab results which fully specify the normal range and the percentage of medication orders which fully specify the ingredient, strength and dose form. Historical and current PCORnet data checks are available on the PCORnet website [13].

## Conclusion

When performing research within a DRN, the data stay local, and analyses are distributed to network participants. Since it is generally not possible for study investigators to examine the patient-level data at each network partner to look for anomalies, it is crucial that the underlying data in a DataMart be of high quality. A data curation process like the one developed for PCORnet can be used to ensure a foundational level of data quality. Individual studies are expected to perform additional checks around the variables or outcomes of interest, but the foundational data curation process allows network partners to identify and fix global issues.

The high level of foundational data quality in PCORnet allows for the rapid execution of queries that can be used as the basis for studies and more specific data quality checks. The iterative nature of the data curation process allows PCORnet to gradually increase the baseline level of quality across the network by adding new checks or tightening existing thresholds over time, while resources like the Implementation Guidance help reduce the variability in practice across the network. These activities help increase the transparency and reproducibility of analyses within PCORnet and can serve as a model for other DRNs.

## Additional Files

The additional files for this article can be found as follows:

10.5334/egems.199.s1 Table 1A. Demographic Summary.

10.5334/egems.199.s1 Table 1B. PCORnet Dashboard Metrics.

10.5334/egems.199.s1 Table 1C. Height, Weight, and Body Mass Index.

10.5334/egems.199.s1 Table 1D. Records, Patients, Encounters, and Date Ranges by Table.

10.5334/egems.199.s1 Table 1E. Records Per Table By Encounter Type.

10.5334/egems.199.s1 Table 1F. Records Per Table By Year.

10.5334/egems.199.s1 Table 1G. Date Obfuscation or Imputation.

10.5334/egems.199.s1 Table IIA. Primary Key Definitions.

10.5334/egems.199.s1 Table IIB. Values Outside of CDM Specifications.

10.5334/egems.199.s1 Table IIC. Non-Permissible Missing Values.

10.5334/egems.199.s1 Table IIIA. Future Dates.

10.5334/egems.199.s1 Table IIIB. Records With Extreme Values.

10.5334/egems.199.s1 Table IVA. Diagnosis Records Per Encounter, Overall and by Encounter Type.

10.5334/egems.199.s1 Chart IVA. Diagnosis Records Per Encounter by Admit Date and Encounter Type, 2010-Present.

10.5334/egems.199.s1 Table IVB. Procedure Records Per Encounter, Overall and by Encounter Type.

10.5334/egems.199.s1 Chart IVB. Procedure Records Per Encounter by Admit Date and Encounter Type, 2010-Present.

10.5334/egems.199.s1 Table IVC. Missing or Unknown Values.

10.5334/egems.199.s1 Chart IA. Vital Measures by Measurement Date, 2010-Present.

10.5334/egems.199.s1 Chart IB. Trend in Encounters by Admit Date and Encounter Type, 2010-Present.

10.5334/egems.199.s1 Chart IC. Trend in Institutional Encounters by Discharge Date and Encounter Type, 2010-Present.
