# Supplementary material for: Evaluating Foundational Data Quality in the National Patient-Centered Clinical Research Network (PCORnet®)
Source: EGEMS (Wash DC). 2018 Apr 13;6(1):3. doi: 10.5334/egems.199 (PMC5983028; doi:10.5334/egems.199)
Supplement: Table 1A. — Demographic Summary. [file egems-6-1-199-s1.pdf]

Sample PCORnet Cycle 1 Empirical Data Characterization Report

This report is derived from a DataMart's responses to the Data Characterization Query Package. Please refer to the Work Plan for details about the source tables.
DataMart: N/A
Response Date: N/A
Low Cell Count Threshold: N/A

Section	Table	Table Description	Data Check(s)
Section I: Descriptive Information and Analysis Considerations	Table IA	Demographic Summary	n/a
	Table IB	PCORnet Dashboard Metrics	n/a
	Table IC	Height, Weight and Body Mass Index (BMI)	n/a
	Table ID	Records, Patients, Encounters, and Date Ranges by Table	4.01, 4.02, 4.03
	Chart IA	Vital Measures by Measurement Date, 2010-Present	n/a
	Table IE	Records Per Table By Encounter Type	4.04
	Table IF	Records Per Table By Year	n/a
	Chart IB	Trend in Encounters by Admit Date and Encounter Type, 2010-Present	n/a
	Chart IC	Trend in Institutional Encounters by Discharge Date and Encounter Type, 2010-Present	n/a
	Table IG	Date Obfuscation or Imputation	4.05
Section II: Data Model Conformance	Table IIA	Primary Key Definitions	1.05
	Table IIB	Values Outside of CDM Specifications	1.06
	Table IIC	Non-Permissible Missing Values	1.07
Section III: Data Plausibility	Table IIIA	Future Dates	2.01
	Table IIIB	Records With Extreme Values	2.02
Section IV: Data Completeness	Table IVA	Diagnosis Records Per Encounter, Overall and by Encounter Type	3.01
	Chart IVA	Diagnosis Records Per Encounter by Admit Date and Encounter Type, 2010-Present	n/a
	Table IVB	Procedure Records Per Encounter, Overall and by Encounter Type	3.02
	Chart IVB	Procedure Records Per Encounter by Admit Date and Encounter Type, 2010-Present	n/a
	Table IVC	Missing or Unknown Values	3.03, 3.04

Response date is the date the data characterization query package was run, not the date the results were received by the DRN OC. Cell counts below the low-cell count threshold will be displayed as 'BT' (below threshold) and treated as zeroes (0s).

Table IA. Demographic Summary

This table contains general descriptive information about the patients in the DEMOGRAPHIC table. These patients may or may not be represented in other CDM tables.

	N	%	Source table
Patients	1,132,186		DEM_L3_N
Age			DEM_L3_AGEYRSDIST1
Mean	42		
Median	40		
Age group			DEM_L3_AGEYRSDIST2
0-18	200,924	17.7	
19-64	732,213	64.6	
65+	197,837	17.5	
Missing, NI, UN or OT	1,212	0.1	
Hispanic			DEM_L3_HISPDIST
N (No)	265,498	23.5	
R (Refused)	0	0.0	
Y (Yes)	65,357	5.8	
Missing, NI, UN or OT	801,331	70.8	
Sex			DEM_L3_SEXDIST
A (Ambiguous)	0	0.0	
F (Female)	586,496	51.8	
M (Male)	544,340	48.1	
Missing, NI, UN or OT	1,350	0.1	

Table excludes records with values outside of CDM specifications.
Cell counts below the low-cell count threshold are displayed as 'BT' and treated as zeroes (0s). Sums which include 'BT' values are marked with an asterisk (*).
If applicable, data check exceptions are highlighted.

Table IA. Demographic Summary (continued)

	N	%	Source table
Race			DEM_L3_RACEDIST
01 (American Indian or Alaska Native)	8,112	0.7	
02 (Asian)	41,983	3.7	
03 (Black or African American)	29,691	2.6	
04 (Native Hawaiian or Other Pacific Islander)	7,382	0.7	
05 (White)	643,830	56.9	
06 (Multiple Race)	0	0.0	
07 (Refuse to answer)	0	0.0	
Missing, NI, UN or OT	401,188	35.4	

Table excludes records with values outside of CDM specifications.
Cell counts below the low-cell count threshold are displayed as 'BT' and treated as zeroes (0s). Sums which include 'BT' values are marked with an asterisk (*).
If applicable, data check exceptions are highlighted.

Table IB. PCORnet Dashboard Metrics

This table contains the PCORnet Dashboard Metrics which are derived from the data characterization query responses.

Metric	Metric Description	Result	Source table
Unique patients	Number of unique patients with at least 1 encounter	835,112	ENC_L3_N
Unique encounters	Number of unique encounters	47,786,158	ENC_L3_N
Potential pool of patients for observational studies	Number of unique patients with at least 1 ED, EI, IP, or AV encounter within the past 5 years	749,643	ENC_L3_DASH2
Potential pool of patients for trials	Number of unique patients with at least 1 ED, EI, IP, or AV encounter within the past 1 year	434,053	ENC_L3_DASH2
Unique patients with encounters and (a) vital signs, (b) diagnoses, and (c) both	Number of unique patients with VITAL and ENCOUNTER records	564,710	VIT_L3_DASH1
	Number of unique patients with DIAGNOSIS and ENCOUNTER records	780,174	DIA_L3_DASH1
	Number of unique patients with DIAGNOSIS, VITAL and ENCOUNTER records	562,202	XTBL_L3_DASH1

Table excludes records with values outside of CDM specifications.
Cell counts below the low-cell count threshold are displayed as 'BT' and treated as zeroes (0s). Sums which include 'BT' values are marked with an asterisk (*).
If applicable, data check exceptions are highlighted.

Table IC. Height, Weight, and Body Mass Index

This table contains descriptive statistics and frequencies of VITAL table measurements.

	Result	%	Source table
Height measurements			VIT_L3_HT_DIST
Records	3,690,854		
Height (inches), mean	63		
Height (inches), median	65		
Weight measurements			VIT_L3_WT_DIST
Records	7,075,486		
Weight (lbs.), mean	166		
Weight (lbs.), median	167		
Body Mass Index (BMI) measurements			VIT_L3_BMI
Records	5,380,184		
BMI <=25	1,945,303	36.2	
BMI 26-30	1,557,050	28.9	
BMI >=31	1,877,831	34.9	

Table excludes records with null or missing values.
Cell counts below the low-cell count threshold are displayed as 'BT' and treated as zeroes (0s). Sums which include 'BT' values are marked with an asterisk (*).

Table ID. Records, Patients, Encounters, and Date Ranges by Table

This table contains summary counts by table and supports Data Checks 4.01, 4.02, and 4.03. When possible, DataMarts should include data from no later than 2010 to the present. Since the date range may be affected by outlier values the percentage of records prior to January 2010 is also displayed.

	DEMOGRAPHIC	ENROLLMENT	ENCOUNTER	DIAGNOSIS	PROCEDURES	VITAL
**Records**						
N	3,421,157	850,086	76,452,217	75,437,911	49,028,003	62,251,061
**Patients**						
N	3,421,157	850,086	3,337,171	3,314,844	1,868,195	2,828,652
**Encounters**						
N			76,452,217	36,783,996	18,162,276	20,676,514
**Date Range**						
Field	BIRTH_DATE	ENR_START_DATE	ADMIT_DATE	ADMIT_DATE	ADMIT_DATE	MEASURE_DATE
Minimum	1841_12	1860_04	1906_04	1979_11	1980_09	1860_04
Maximum	2015_09	2015_06	2015_09	2015_09	2015_09	2015_09
% of records before Jan 2010	76.0	52.7	34.0	26.4	29.9	17.6
**Source table(s)**	DEM_L3_N; XTBL_L3_DATES	ENR_L3_N; XTBL_L3_DATES	ENC_L3_N; XTBL_L3_DATES	DIA_L3_N; XTBL_L3_DATES	PRO_L3_N; XTBL_L3_DATES	VIT_L3_N; XTBL_L3_DATES

Data check exceptions are highlighted.

Chart IA. Vital Measures by Measurement Date, 2010-Present

This chart illustrates changes over time in the number of records in the VITAL table. Monthly record counts for specific vital measures are not available.

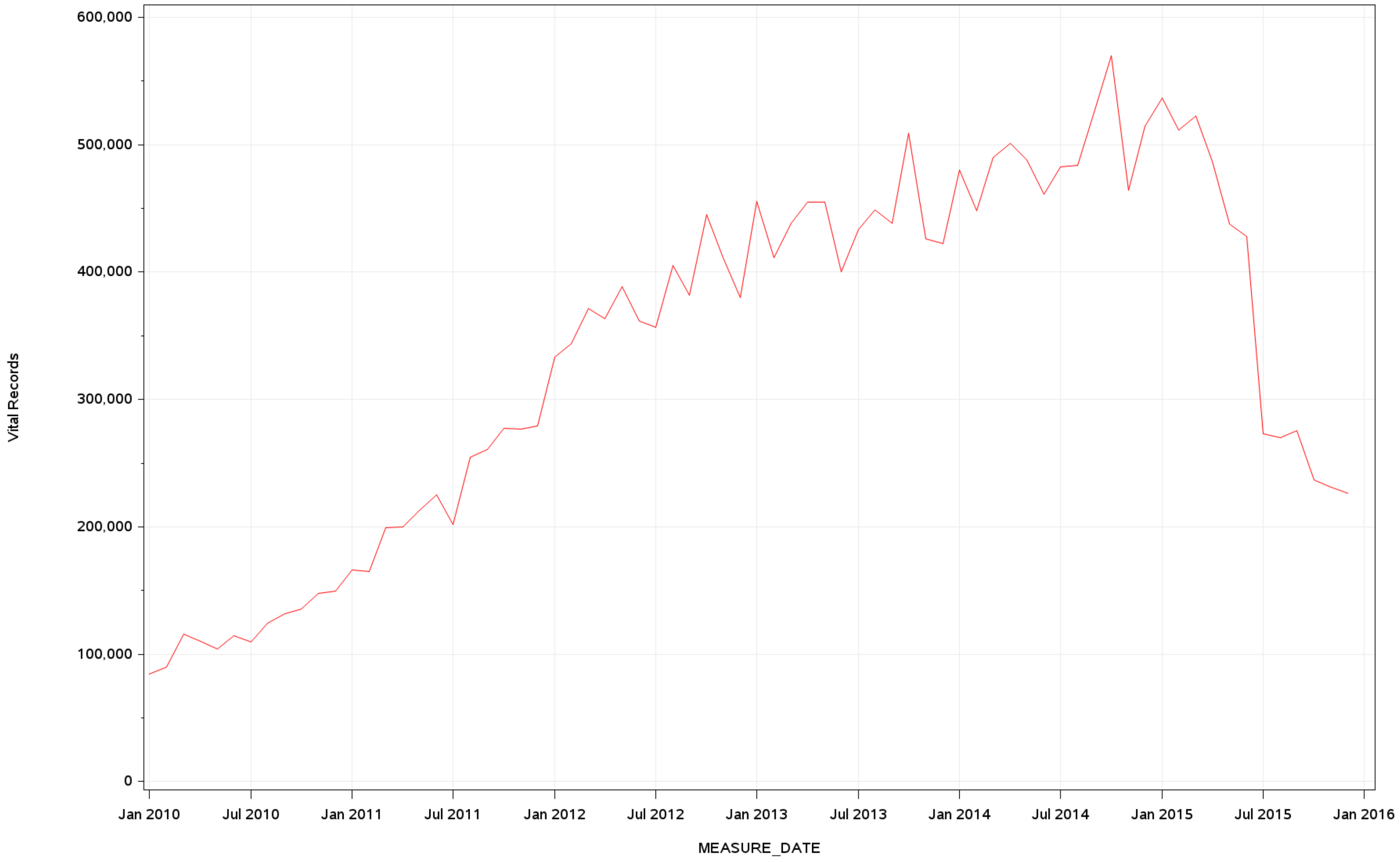

Data check exceptions are highlighted.

Table IE. Records Per Table By Encounter Type

This table contains record counts by encounter type for the ENCOUNTER, DIAGNOSIS, and PROCEDURES tables and supports Data Check 4.04. The presence of ambulatory (AV), inpatient (IP or EI), and emergency department (ED or EI) encounters is not required but may be important for some research studies.

	ENCOUNTER		DIAGNOSIS		PROCEDURES	
Encounter Type	N	%	N	%	N	%
AV (Ambulatory Visit)	267,151	2.0	26,756,870	76.8	44,372,674	62.2
ED (Emergency Dept)	620,593	4.6	2,139,820	6.1	3,841,255	5.4
EI (ED to IP Stay)	0		0		0	
IP (Inpatient Hospital Stay)	372,384	2.7	5,935,177	17.0	23,117,270	32.4
IS (Non-acute Institutional Stay)	0		0		0	
OA (Other Ambulatory Visit)	0		0		0	
Missing, NI, UN or OT	12,371,181	90.8	14,192	0.0	57,786	0.1
Total	13,631,309		34,846,059		71,388,985	
Source table	ENC_L3_ENCTYPE		DIA_L3_ENCTYPE		PRO_L3_ENCTTPE	

Table excludes records with values outside of CDM specifications.
Cell counts below the low-cell count threshold are displayed as 'BT' and treated as zeroes (0s). Sums which include 'BT' values are marked with an asterisk (*).
If applicable, data check exceptions are highlighted.

Table IF. Records Per Table By Year

This table contains the number of records in the ENROLLMENT, ENCOUNTER, DIAGNOSIS, PROCEDURES, and VITAL tables. All years prior to 2010 and after 2015 are combined.

	ENROLLMENT		ENCOUNTER		DIAGNOSIS		PROCEDURES		VITAL	
Year	N	%	N	%	N	%	N	%	N	%
<2010	3,868	0	4,472	0	16,772	0	3,784	0		
2010	453,190	37	5,132,775	15	9,579,702	11	4,828,419	17		
2011	201,157	16	6,159,793	18	11,466,005	13	5,837,139	20	47,644	0
2012	166,524	14	5,813,230	17	12,637,481	14	5,252,536	18	2,941,904	10
2013	137,172	11	5,890,768	17	17,257,783	19	4,770,222	17	8,397,870	30
2014	142,512	12	5,952,931	18	19,622,265	22	4,534,851	16	9,151,199	33
2015	117,233	10	4,891,909	14	17,926,884	20	3,628,841	13	7,496,363	27
>=2016	0	0								
Total	1,221,656		33,845,878		88,506,892		28,855,792		28,034,980	
Source	ENR_L3_ENR_YM		ENC_L3_ADATE_Y		DIA_L3_ADATE_Y		PRO_L3_ADATE_Y		VIT_L3_MDATE_Y	

Table excludes records with null or missing values.
Cell counts below the low-cell count threshold are displayed as 'BT' and treated as zeroes (0s). Sums which include 'BT' values are marked with an asterisk (*).
Year=ENCOUNTER.ADMIT_DATE, ENROLLMENT.ENR_START_DATE, DIAGNOSIS.ADMIT_DATE, PROCEDURES.ADMIT_DATE, or VITAL.MEASURE_DATE.

Chart IB. Trend in Encounters by Admit Date and Encounter Type, 2010-Present

This chart illustrates relative changes over time in the number of records per encounter type found in the ENCOUNTER table. Monthly record counts for each encounter type were standardized over the period shown to have a mean of 0 and a standard deviation of 1. The y-axis therefore reflects the deviation in each month's count from the average over the whole time period. A value above the center line of 0 indicates an above-average number of encounters; a value below the center line of 0 indicates a below-average number of encounters. Incomplete data in recent months or the introduction of new encounter types may contribute to fluctuations.

AV (Ambulatory Visit)

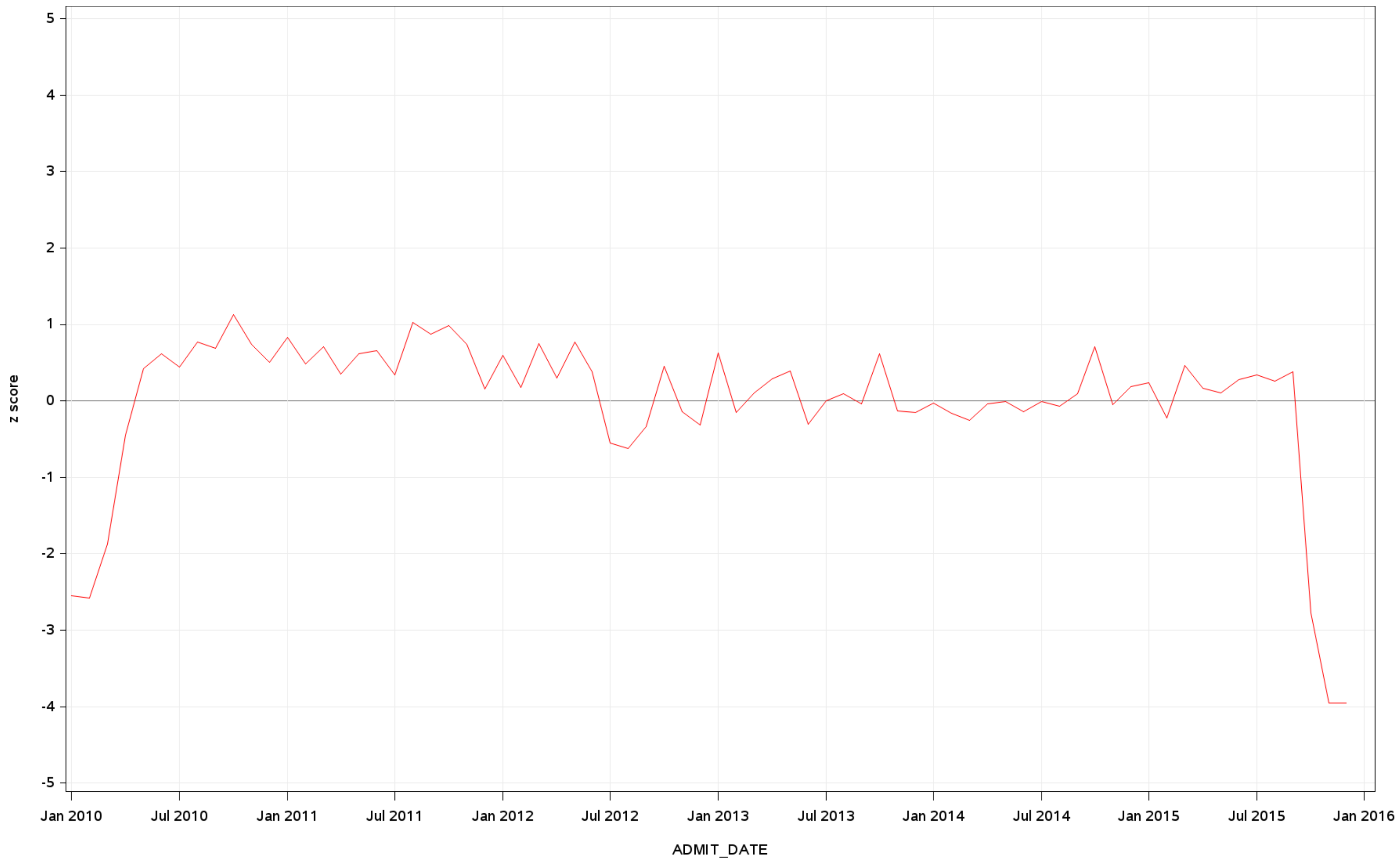

Chart IB. Trend in Encounters by Admit Date and Encounter Type, 2010-Present

This chart illustrates relative changes over time in the number of records per encounter type found in the ENCOUNTER table. Monthly record counts for each encounter type were standardized over the period shown to have a mean of 0 and a standard deviation of 1. The y-axis therefore reflects the deviation in each month's count from the average over the whole time period. A value above the center line of 0 indicates an above-average number of encounters; a value below the center line of 0 indicates a below-average number of encounters. Incomplete data in recent months or the introduction of new encounter types may contribute to fluctuations.

ED (Emergency Dept)

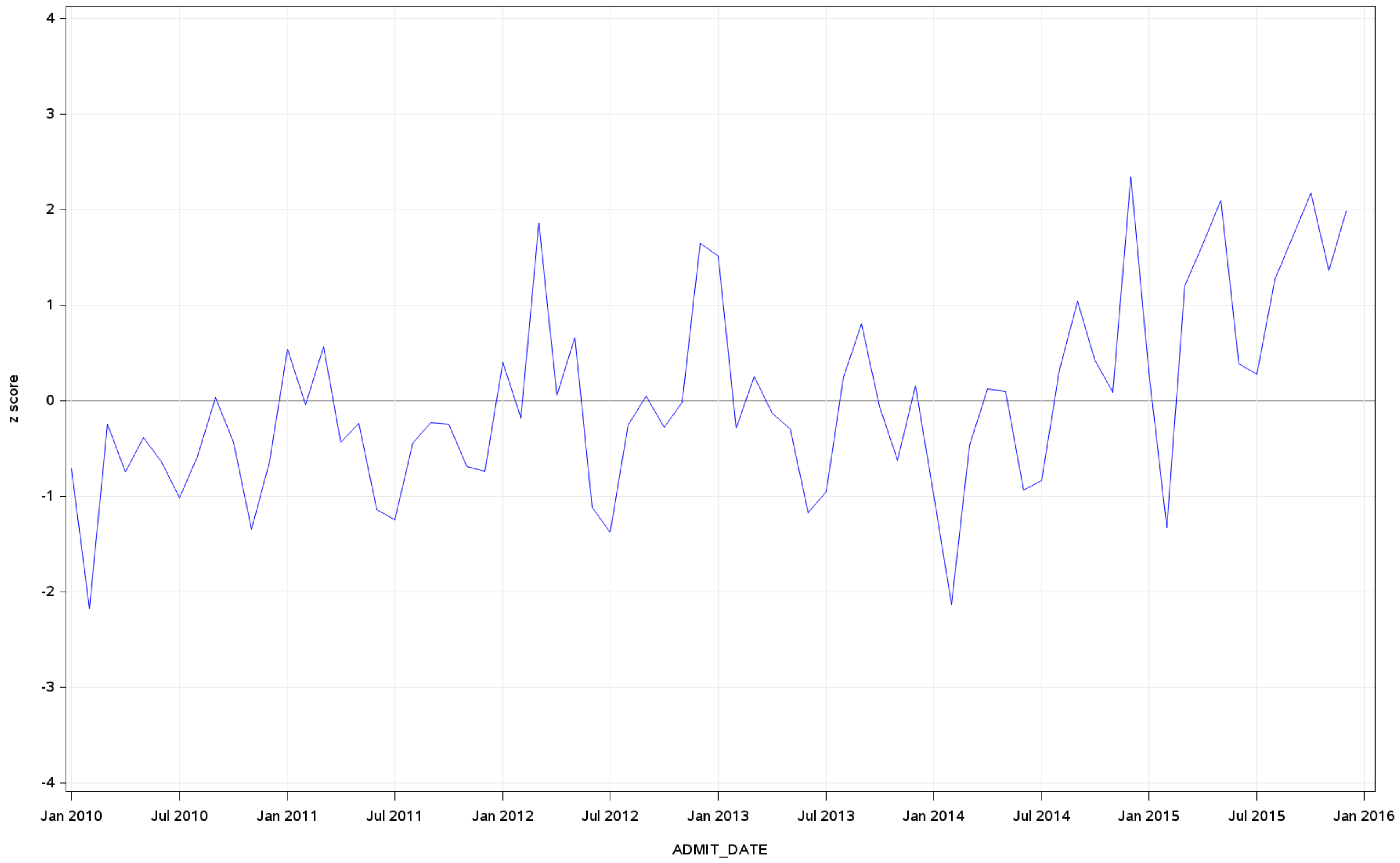

Chart IB. Trend in Encounters by Admit Date and Encounter Type, 2010-Present

This chart illustrates relative changes over time in the number of records per encounter type found in the ENCOUNTER table. Monthly record counts for each encounter type were standardized over the period shown to have a mean of 0 and a standard deviation of 1. The y-axis therefore reflects the deviation in each month's count from the average over the whole time period. A value above the center line of 0 indicates an above-average number of encounters; a value below the center line of 0 indicates a below-average number of encounters. Incomplete data in recent months or the introduction of new encounter types may contribute to fluctuations.

IP (Inpatient Hospital Stay)

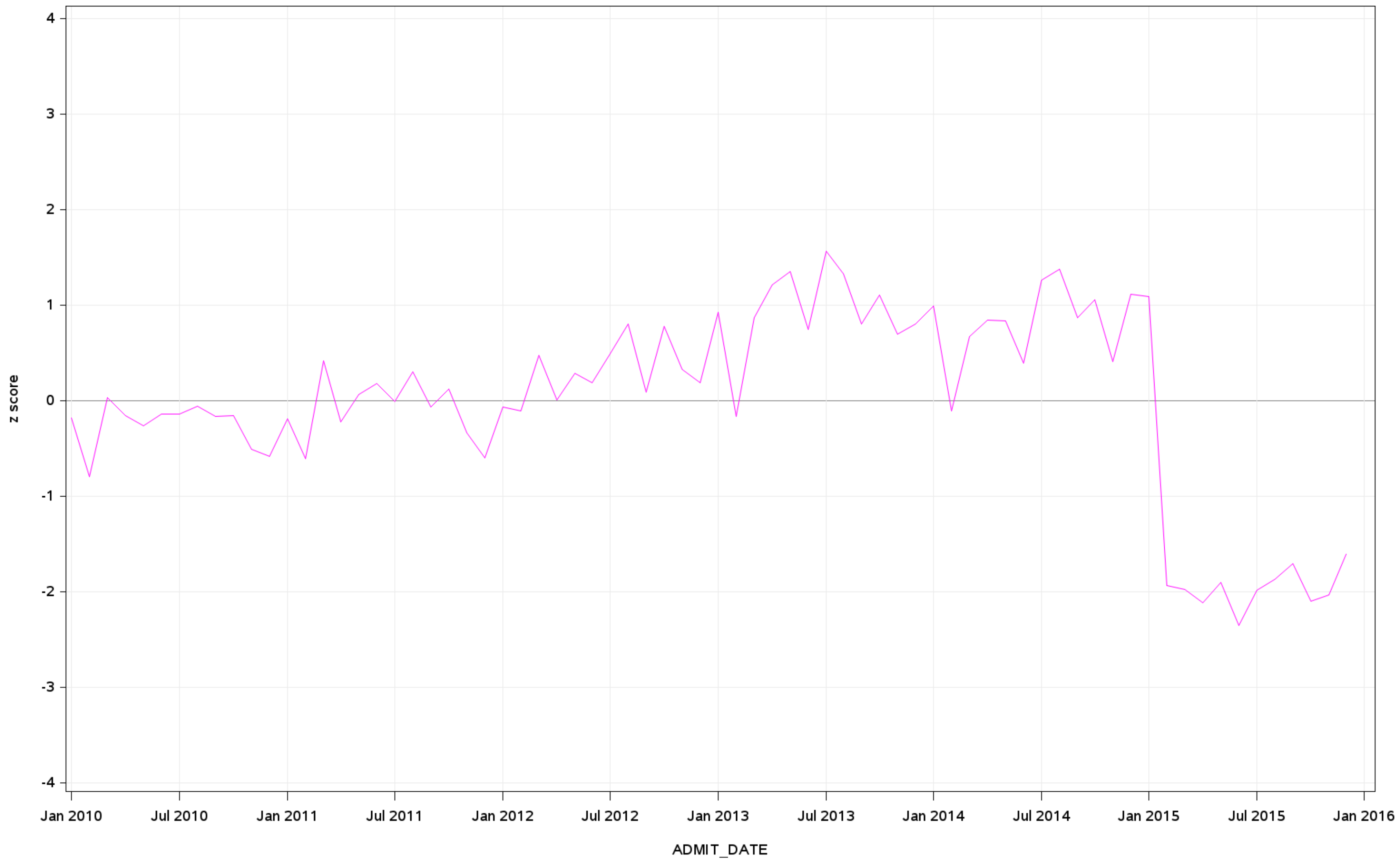

Chart IC. Trend in Institutional Encounters by Discharge Date and Encounter Type, 2010-Present

This chart illustrates relative changes over time in the number of records per institutional (IP, IS, or EI) encounter type found in the ENCOUNTER table. Monthly record counts for each encounter type were standardized over the period shown to have a mean of 0 and a standard deviation of 1. The y-axis therefore reflects the deviation in each month's count from the average over the whole time period. A value above the center line of 0 indicates an above-average number of encounters; a value below the center line of 0 indicates a below-average number of encounters. Incomplete data in recent months or the introduction of new encounter types may contribute to fluctuations.

IP (Inpatient Hospital Stay)

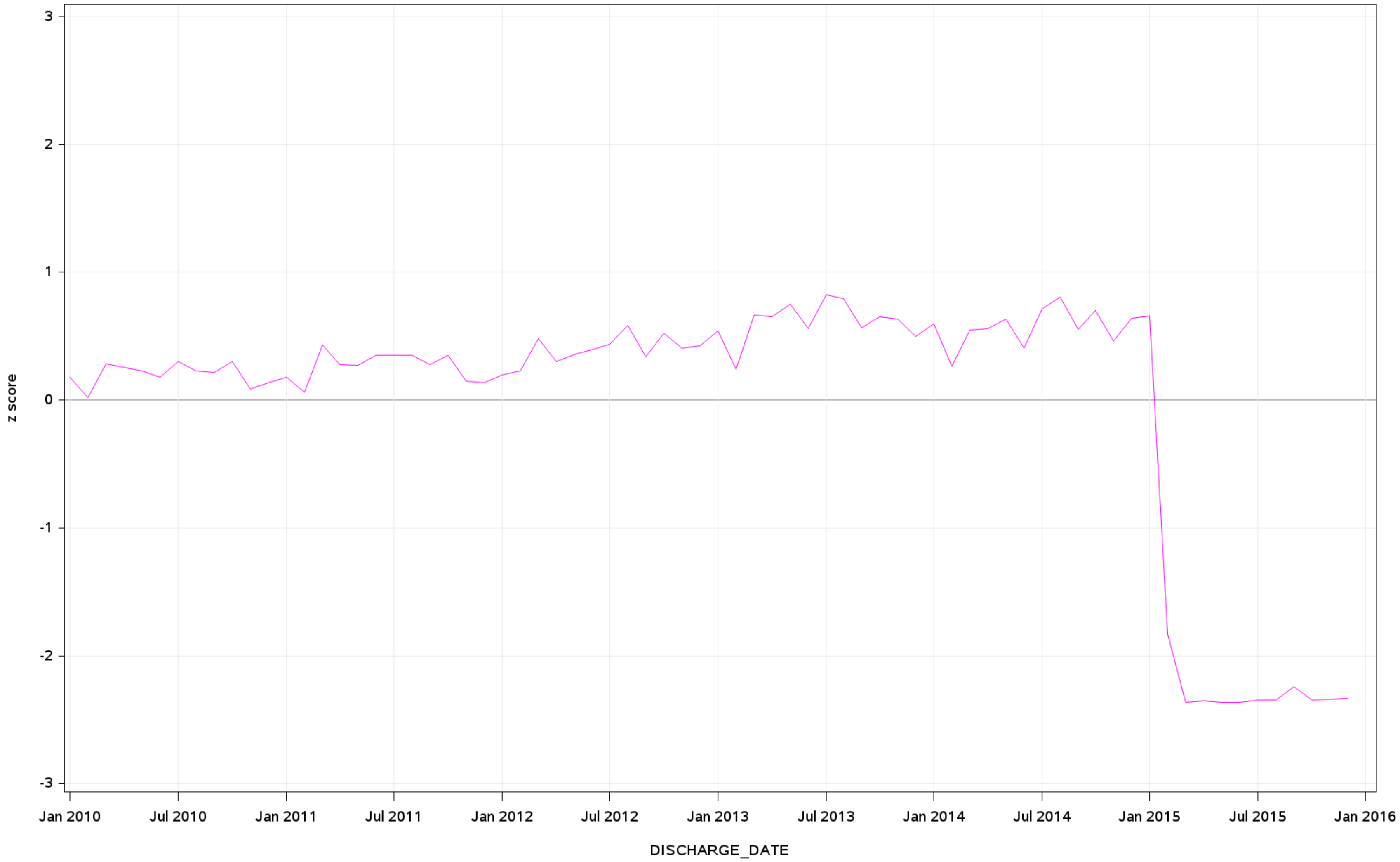

Table IG. Date Obfuscation or Imputation

This table contains information about the presence of date obfuscation or imputation for relevant fields in the DEMOGRAPHIC, ENROLLMENT, ENCOUNTER, DIAGNOSIS, PROCEDURES, or VITAL tables and supports Data Check 4.05. Imputed or obfuscated dates are permissible but are important to consider when interpreting results.

Table	Field	Date Obfuscation or Imputation	Source table
HARVEST	BIRTH_DATE_MGMT	No	XTBL_L3_METADATA
HARVEST	ENR_START_DATE_MGMT	No	XTBL_L3_METADATA
HARVEST	ENR_END_DATE_MGMT	No	XTBL_L3_METADATA
HARVEST	ADMIT_DATE_MGMT	No	XTBL_L3_METADATA
HARVEST	DISCHARGE_DATE_MGMT	No	XTBL_L3_METADATA
HARVEST	PX_DATE_MGMT	No	XTBL_L3_METADATA
HARVEST	MEASURE_DATE_MGMT	No	XTBL_L3_METADATA

Table excludes records with null or missing values.
Cell counts below the low-cell count threshold are displayed as 'BT' and treated as zeroes (0s). Sums which include 'BT' values are marked with an asterisk (*).
Year=ENCOUNTER.ADMIT_DATE, ENROLLMENT.ENR_START_DATE, DIAGNOSIS.ADMIT_DATE, PROCEDURES.ADMIT_DATE, or VITAL.MEASURE_DATE.

Table IIA. Primary Key Definitions

This table shows the required primary key definitions for the DEMOGRAPHIC, ENROLLMENT, ENCOUNTER, DIAGNOSIS, PROCEDURES, VITAL and HARVEST tables and supports Data Check 1.05. The ETL will need to be modified to address any area of non-conformance.

Table	CDM specifications for primary keys	Table conforms to specifications?	Source table
DEMOGRAPHIC	PATID is unique	Yes	DEM_L3_N
ENROLLMENT	PATID + ENR_START_DATE+ENR_BASIS is unique	Yes	ENR_L3_N
ENCOUNTER	ENCOUNTERID is unique	Yes	ENC_L3_N
DIAGNOSIS	DIAGNOSISID is unique	Yes	DIA_L3_N
PROCEDURES	PROCEDURESID is unique	Yes	PRO_L3_N
HARVEST	NETWORKID+DATAMARTID is unique	Yes	XTBL_L3_METADATA

Table excludes records with values outside of CDM specifications.
Cell counts below the low-cell count threshold are displayed as 'BT' and treated as zeroes (0s). Sums which include 'BT' values are marked with an asterisk (*).
If applicable, data check exceptions are highlighted.

Table IIB. Values Outside of CDM Specifications

This table lists all fields with pre-defined value lists in the DEMOGRAPHIC, ENROLLMENT, ENCOUNTER, DIAGNOSIS, PROCEDURES, and VITAL tables and supports Data Check 1.06. The ETL will need to be modified to address any area of non-conformance.

Table	Field	Number of records with values outside of specifications	Source table
DEMOGRAPHIC	SEX	0	DEM_L3_SEXDIST
DEMOGRAPHIC	HISPANIC	0	DEM_L3_HISPDIST
DEMOGRAPHIC	RACE	0	DEM_L3_RACEDIST
ENROLLMENT	ENR_BASIS	0	ENR_L3_BASEDIST
ENCOUNTER	ENC_TYPE	0	ENC_L3_ENCTYPE
ENCOUNTER	DISCHARGE_DISPOSITION	0	ENC_L3_DISDISP
ENCOUNTER	DISCHARGE_STATUS	0	ENC_L3_DISSTAT
ENCOUNTER	DRG_TYPE	0	ENC_L3_DRG_TYPE
ENCOUNTER	ADMITTING_SOURCE	0	ENC_L3_ADMSRC
DIAGNOSIS	ENC_TYPE	0	DIA_L3_ENCTYPE
DIAGNOSIS	DX_TYPE	0	DIA_L3_DXTYPE_DXSOURCE
DIAGNOSIS	DX_SOURCE	0	DIA_L3_DXSOURCE
DIAGNOSIS	PDX	0	DIA_L3_PDX
PROCEDURES	ENC_TYPE	0	PRO_L3_ENCTYPE
PROCEDURES	PX_SOURCE	0	PRO_L3_PXSOURCE

Cell counts below the low-cell count threshold are displayed as 'BT' and treated as zeroes (0s). Sums which include 'BT' values are marked with an asterisk (*).
Data check exceptions are highlighted.

Table IIB. Values Outside of CDM Specifications (continued)

Table	Field	Number of records with values outside of specifications	Source table
HARVEST	DATAMART_PLATFORM	0	XTBL_L3_METADATA
HARVEST	DATAMART_CLAIMS	0	XTBL_L3_METADATA
HARVEST	DATAMART_EHR	0	XTBL_L3_METADATA
HARVEST	BIRTH_DATE_MGMT	0	XTBL_L3_METADATA
HARVEST	ENR_START_DATE_MGMT	0	XTBL_L3_METADATA
HARVEST	ENR_END_DATE_MGMT	0	XTBL_L3_METADATA
HARVEST	ADMIT_DATE_MGMT	0	XTBL_L3_METADATA
HARVEST	DISCHARGE_DATE_MGMT	0	XTBL_L3_METADATA
HARVEST	PX_DATE_MGMT	0	XTBL_L3_METADATA

Cell counts below the low-cell count threshold are displayed as 'BT' and treated as zeroes (0s). Sums which include 'BT' values are marked with an asterisk (*).
Data check exceptions are highlighted.

Table IIC. Non-Permissible Missing Values

This table contains the number of records with missing values for all fields which are required to be populated and supports Data Check 1.07. The ETL will need to be modified to address any area of non-conformance.

Table	Field	Number of records with missing values	Source table
DEMOGRAPHIC	PATID	0	DEM_L3_N
ENROLLMENT	PATID	0	ENR_L3_N
ENROLLMENT	ENR_START_DATE	0	ENR_L3_N
ENROLLMENT	ENR_BASIS	0	ENR_L3_BASEDIST
ENCOUNTER	PATID	0	ENC_L3_N
ENCOUNTER	ENCOUNTERID	0	ENC_L3_N
ENCOUNTER	ADMIT_DATE	0	ENC_L3_ADATE_Y
ENCOUNTER	ENC_TYPE	0	ENC_L3_ENCTYPE
DIAGNOSIS	DIAGNOSISID	0	DIA_L3_N
DIAGNOSIS	PATID	0	DIA_L3_N
DIAGNOSIS	ENCOUNTERID	0	DIA_L3_N
DIAGNOSIS	DX	0	DIA_L3_DX
DIAGNOSIS	DX_TYPE	0	DIA_L3_DXTYPE_ENCTYPE
DIAGNOSIS	DX_SOURCE	0	DIA_L3_DXSOURCE
PROCEDURES	PROCEDURESID	0	PRO_L3_N
PROCEDURES	PATID	0	PRO_L3_N
PROCEDURES	ENCOUNTERID	0	PRO_L3_N
PROCEDURES	PX	0	PRO_L3_PX
PROCEDURES	PX_TYPE	0	PRO_L3_PXTYPE_ENCTYPE
HARVEST	NETWORKID	0	XTBL_L3_METADATA
HARVEST	DATAMARTID	0	XTBL_L3_METADATA

Data check exceptions are highlighted.

Table IIIA. Future Dates

This table illustrates the percentage of records with future dates for all date fields in the DEMOGRAPHIC, ENROLLMENT, ENCOUNTER, DIAGNOSIS, PROCEDURES, VITAL and HARVEST tables and supports Data Check 2.01. Future dates may be attributable to data entry errors in the source data or ETL errors such as including scheduled appointments in the ENCOUNTER table.

		Records with future dates			
Table	Field	Numerator	Denominator	%	Source table(s)
DEMOGRAPHIC	BIRTH_DATE	0	3,326,773	0.00	XTBL_L3_DATES; DEM_L3_AGEYRSDIST1
ENROLLMENT	ENR_START_DATE	0	2,506,074	0.00	XTBL_L3_DATES; ENR_L3_DIST_START
ENROLLMENT	ENR_END_DATE	0	2,506,074	0.00	XTBL_L3_DATES; ENR_L3_DIST_END
ENCOUNTER	ADMIT_DATE	0	228,531,286*	0.00	XTBL_L3_DATES; ENC_L3_ADATE_Y
ENCOUNTER	DISCHARGE_DATE	0	74,358,017	0.00	XTBL_L3_DATES; ENC_L3_DDATE_Y
DIAGNOSIS	ADMIT_DATE	0	497,301,023*	0.00	XTBL_L3_DATES; DIA_L3_ADATE_Y
PROCEDURES	ADMIT_DATE	0	621,128,489*	0.00	XTBL_L3_DATES; PRO_L3_ADATE_Y
PROCEDURES	PX_DATE	0	621,036,216*	0.00	XTBL_L3_DATES; PRO_L3_PXDATE_Y
HARVEST	REFRESH_DEMOGRAPHIC_DATE	0			XTBL_L3_METADATA
HARVEST	REFRESH_ENROLLMENT_DATE	0			XTBL_L3_METADATA
HARVEST	REFRESH_ENCOUNTER_DATE	0			XTBL_L3_METADATA
HARVEST	REFRESH_DIAGNOSIS_DATE	0			XTBL_L3_METADATA
HARVEST	REFRESH_PROCEDURES_DATE	0			XTBL_L3_METADATA
HARVEST	REFRESH_VITAL_DATE	0			XTBL_L3_METADATA
HARVEST	REFRESH_MAX	0			XTBL_L3_METADATA

Denominator excludes null or missing values.
Cell counts below the low-cell count threshold are displayed as 'BT' and treated as zeroes (0s).

Table IIIB. Records With Extreme Values

This table lists the number of records in the lowest or highest categories of age, height, weight, diastolic blood pressure, and/or systolic blood pressure and supports Data Check 2.02. A high percentage of records in these categories may indicate incorrect measurement units.

		Data Check Parameters			Records with values in the lowest category		Records with values in the highest category		
Table	Field	Low	High	Records	N	%	N	%	Source table
DEMOGRAPHIC	AGE (derived from BIRTH_DATE)	<0 yrs.	>110 yrs.	1,719,513	0		23,343	1.4	DEM_L3_AGEYRSDIST2
VITAL	HT	<0 inches	>=95 inches	1,962,686	0		1,945,700	30.8	VIT_L3_HT
VITAL	WT	<0 lbs.	>350 lbs.	3,903,620	0		292	0.0	VIT_L3_WT
VITAL	DIASTOLIC	<40 mgHg	>120 mgHg	5,681,437	31,391	0.5	2,453	0.0	VIT_L3_DIASTOLIC
VITAL	SYSTOLIC	<40 mgHg	>210 mgHg	5,681,444	1,987	0.0	2,184	0.0	VIT_L3_SYSTOLIC

Table excludes records with values outside of CDM specifications
Cell counts below the low-cell count threshold are displayed as 'BT' and treated as zeroes (0s). Sums which include 'BT' values are marked with an asterisk (*).
Table excludes records with null or missing values.
Data check exceptions are highlighted.

Table IVA. Diagnosis Records Per Encounter, Overall and by Encounter Type

This table displays the average number of diagnoses records per encounter and supports Data Check 3.01. Low number of diagnoses per encounter may be due to source data limitations or incomplete data capture. In general, the number of diagnosis codes should be higher for institutional encounters than for ambulatory encounters.

Encounter Type	DIAGNOSIS records	ENCOUNTER records	Diagnoses Per Encounter	Source table
AV (Ambulatory Visit)	46,287,851	22,365,820	2.07	DIA_L3_ENCTYPE; ENC_L3_ENCTYPE
ED (Emergency Dept)	703,744	215,207	3.27	DIA_L3_ENCTYPE; ENC_L3_ENCTYPE
EI (ED to IP Stay)	0	0		DIA_L3_ENCTYPE; ENC_L3_ENCTYPE
IP (Inpatient Hospital Stay)	10,979,189	5,596,189	1.96	DIA_L3_ENCTYPE; ENC_L3_ENCTYPE
IS (Non-acute Institutional Stay)	310,673	42,099	7.38	DIA_L3_ENCTYPE; ENC_L3_ENCTYPE
OA (Other Ambulatory Visit)	10,430,943	21,830,313	0.48	DIA_L3_ENCTYPE; ENC_L3_ENCTYPE
Missing, NI, UN or OT	1,322,047	6,114,845	0.22	DIA_L3_ENCTYPE; ENC_L3_ENCTYPE
Total	70,034,447	56,164,473	1.25	DIA_L3_ENCTYPE; ENC_L3_ENCTYPE

Cell counts below the low-cell count threshold are displayed as 'BT' and treated as zeroes (0s). Sums which include 'BT' values are marked with an asterisk (*).
These calculations assume that all encounters in DIAGNOSIS are in ENCOUNTER.
Data check exceptions are highlighted.

Chart IVA. Diagnosis Records Per Encounter by Admit Date and Encounter Type, 2010-Present

This chart displays changes over time in the number of diagnosis codes per encounter. Pronounced differences may be due to source data changes including changes in coding practices. Incomplete data in recent months may contribute to fluctuations.

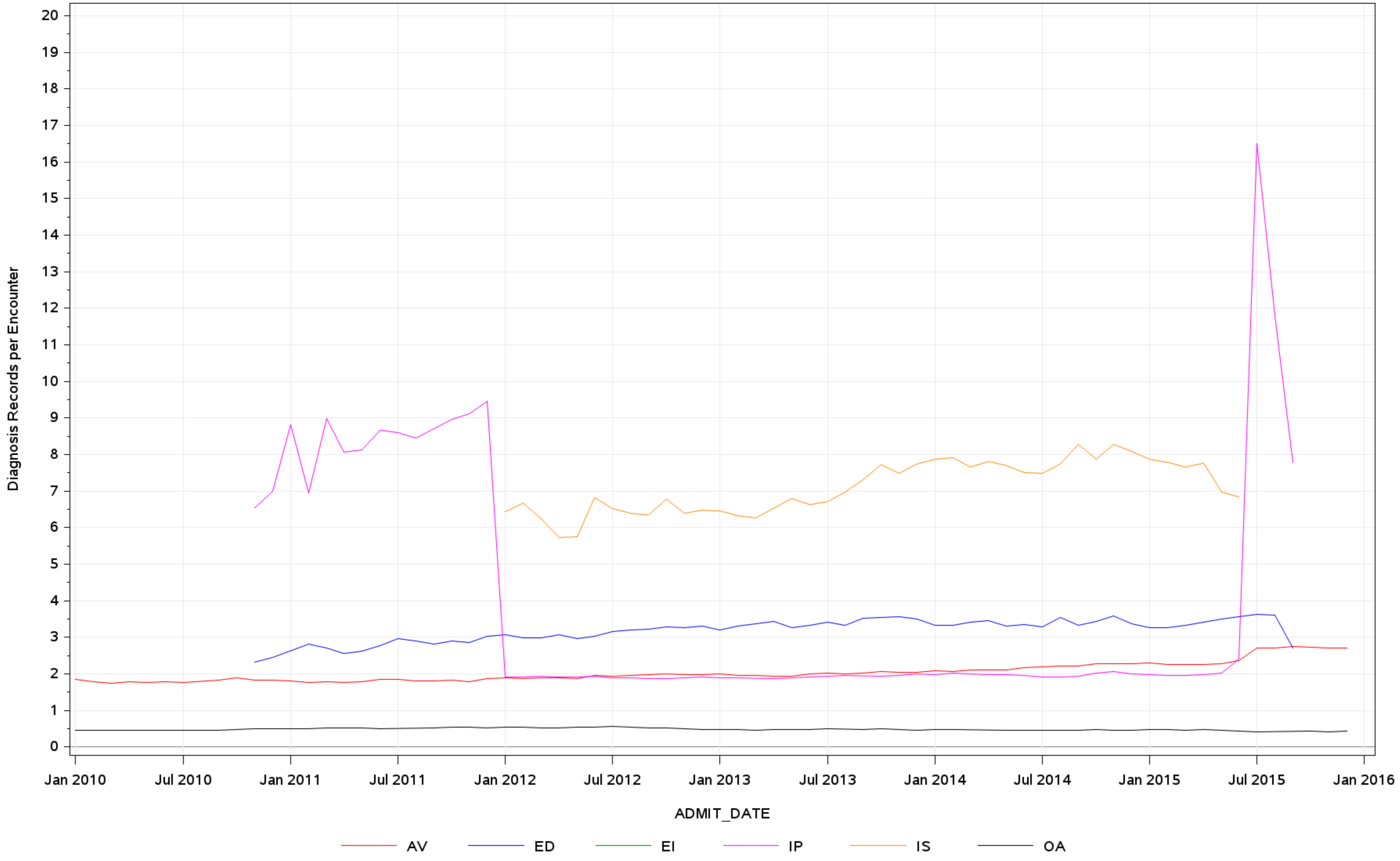

Table IVB. Procedure Records Per Encounter, Overall and by Encounter Type

This table displays the average number of procedure records per encounter and supports Data Check 3.02. Low numbers of procedures per encounter may be due to source data limitations or incomplete data capture.

Encounter Type	PROCEDURES records	ENCOUNTER records	Procedures Per Encounter	Source table
AV (Ambulatory Visit)	36,959,195	46,202,069	0.80	PRO_L3_ENCTYPE; ENC_L3_ENCTYPE
ED (Emergency Dept)	2,485,366	2,819,978	0.88	PRO_L3_ENCTYPE; ENC_L3_ENCTYPE
EI (ED to IP Stay)	0	0		PRO_L3_ENCTYPE; ENC_L3_ENCTYPE
IP (Inpatient Hospital Stay)	7,363,249	1,776,570	4.14	PRO_L3_ENCTYPE; ENC_L3_ENCTYPE
IS (Non-acute Institutional Stay)	0	0		PRO_L3_ENCTYPE; ENC_L3_ENCTYPE
OA (Other Ambulatory Visit)	2,219,763	24,701,546	0.09	PRO_L3_ENCTYPE; ENC_L3_ENCTYPE
Missing, NI, UN or OT	430	952,054	0.00	PRO_L3_ENCTYPE; ENC_L3_ENCTYPE
Total	49,028,003	76,452,217	0.64	PRO_L3_ENCTYPE; ENC_L3_ENCTYPE

Cell counts below the low-cell count threshold are displayed as 'BT' and treated as zeroes (0s). Sums which include 'BT' values are marked with an asterisk (*).
These calculations assume that all encounters in PROCEDURES are in ENCOUNTER.
Data check exceptions are highlighted.

Chart IVB. Procedure Records Per Encounter by Admit Date and Encounter Type, 2010-Present

This chart displays changes over time in the number of procedure codes per encounter. Pronounced differences may be due to source data changes including changes in coding practices. Incomplete data in recent months may contribute to fluctuations.

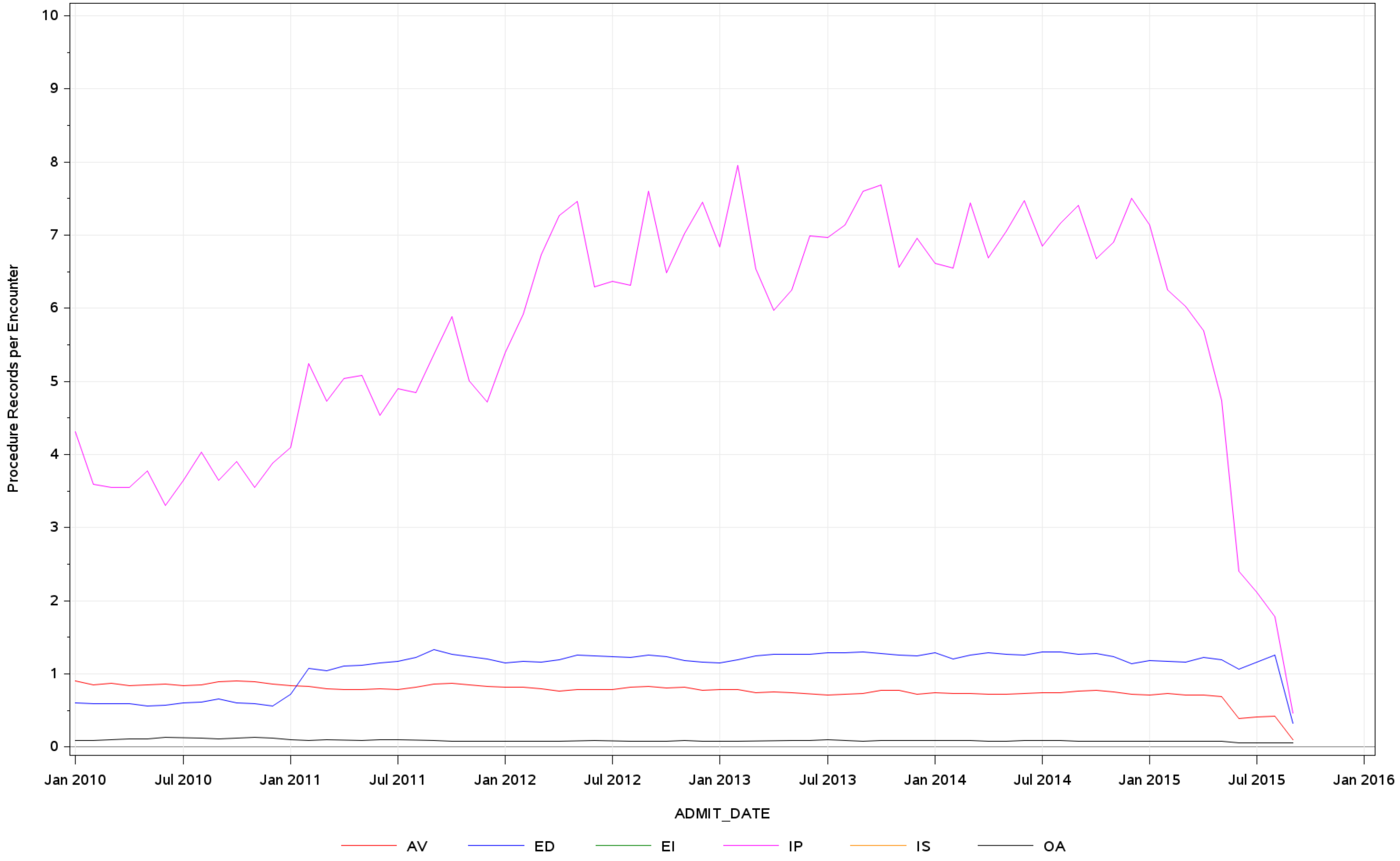

Table IVC. Missing or Unknown Values

This table includes fields in the DEMOGRAPHIC, ENROLLMENT, ENCOUNTER, DIAGNOSIS, PROCEDURES and VITAL tables which are included in the query results and are not required to be populated (see Table IIC for required fields). VITAL measures are not included because the VITAL table structure (1 record per result) does not support missingness assessment. The table depicts the percentage of records with missing or unknown values and supports Data Checks 3.03 and 3.04.

			Records with missing, NI, UN, or OT values			Data Check		
Table	Field	Encounter Type Constraint	Numerator	Denominator	%	Data Check	Threshold	Source
DEMOGRAPHIC	BIRTH_DATE		0	3,421,157		3.03	>=5%	DEM_L3_AGEYRSDIST1
DEMOGRAPHIC	SEX		353*	3,421,157	0.0	3.03	>=5%	DEM_L3_SEXDIST
DEMOGRAPHIC	HISPANIC		547,518	3,421,157	16.0	--	--	DEM_L3_HISPDIST
DEMOGRAPHIC	RACE		691,151	3,421,157	20.2	3.04	>=15%	DEM_L3_RACEDIST
ENROLLMENT	ENR_END_DATE		0	850,086		--	--	ENR_L3_DIST_END
ENCOUNTER	DISCHARGE_DATE	IP, IS, EI	246,507	1,776,378*	13.9	--	--	ENC_L3_ENCTYPE_DDATE_YM
ENCOUNTER	ENC_TYPE		952,054	76,452,217	1.2	--	--	ENC_L3_ENCTYPE
ENCOUNTER	PROVIDERID		28,402,224	76,452,217	37.2	--	--	ENC_L3_N
ENCOUNTER	DISCHARGE_DISPOSITION	IP, IS, EI	1,251,701	1,776,570	70.5	3.04	>=15%	ENC_L3_ENCTYPE_DISDISP
ENCOUNTER	DISCHARGE_STATUS	IP, IS, EI	1,382,582	1,776,554*	77.8	--	--	ENC_L3_ENCTYPE_DISSTAT
ENCOUNTER	DRG	IP, IS, EI	1,556,810	1,771,305*	87.9	--	--	ENC_L3_ENCTYPE_DRG
ENCOUNTER	ADMITTING_SOURCE	IP, IS, EI	1,323,466	1,776,557*	74.5	--	--	ENC_L3_ENCTYPE_ADMRSC
DIAGNOSIS	DX_TYPE		1,077,349	75,437,911	1.4	3.03	>=5%	DIA_L3_DXTYPE_DXSOURCE
DIAGNOSIS	DX_SOURCE		75,437,911	75,437,911	100.0	--	--	DIA_L3_DXSOURCE
DIAGNOSIS	PDX	IP, IS, EI	2,880,050	2,880,050	100.0	3.04	>=15%	DIA_L3_PDX_ENCTYPE
PROCEDURES	PX_DATE		49,028,003	49,028,003	100.0	--	--	PRO_L3_PXDATE_Y
PROCEDURES	PX_TYPE		8,330,619*	49,028,003	17.0	3.03	>=5%	PRO_L3_PXTYPE_ENCTYPE
PROCEDURES	PX_SOURCE		49,028,003	49,028,003	100.0	--	--	PRO_L3_PXSOURCE
VITAL	VITAL_SOURCE		0	62,251,061		3.03	>=5%	VIT_L3_VITAL_SOURCE

Table excludes records with values outside of CDM specifications
Cell counts for non-missing values are displayed as 'BT' and treated as zeroes (0s). Sums which include 'BT' values are marked with an asterisk (*).
The four 'flavors of null' defined in the CDM are combined in this table but details are available in the source tables.
Data check exceptions are highlighted.
